# Unravelling the complexity in achieving the 17 sustainable-development goals

**DOI:** 10.1093/nsr/nwz038

**Published:** 2019-03-15

**Authors:** Bojie Fu, Shuai Wang, Junze Zhang, Zengqian Hou, Jinghai Li

**Affiliations:** 1 State Key Laboratory of Urban and Regional Ecology, Research Center for Eco-Environmental Sciences, Chinese Academy of Sciences, China; 2 State Key Laboratory of Earth Surface Processes and Resource Ecology, Faculty of Geographical Science, Beijing Normal University, China; 3 National Natural Science Foundation of China, China

It is now more than 3 years since the ratification of the 2030 Agenda for Sustainable Development framework [1] but, according to the latest progress report, unless the rate of progress increases, it is challenging that all of the Sustainable Development Goals (SDGs) will be achieved by 2030 [2]. With only 11 years remaining until the 2030 deadline, it has become imperative to develop a plan of action to enable the full agenda to be realized. Specifically, there is an urgent need for a holistic approach to clarify the interrelationships between the 17 SDGs, while also taking into account their complexity and their sometimes mutually reinforcing or conflicting nature. Trying to achieve these goals separately in succession is nonsensical, but pursuing them simultaneously is impractical [3]. The fundamental logic underlying these complex relationships between the goals must be systematically expressed; only then will we be able to fully achieve them.

Many studies have been conducted on the interlinkages among these 17 ‘holistic’ and ‘indivisible’ goals, and they have taken a range of different approaches. These include applying a nexus approach to the various goals [4], investigating the degree of interactions among the different goals [5], employing network analysis [6] and even developing a sustainable-development model [7]. However, these studies have only analysed the synergies and trade-offs among the various goals, and they have usually championed the goals believed to be the most important while often ignoring the importance of others. This situation has made it impossible for policymakers to recommend ways in which to holistically achieve the full set of SDGs.

Here, we regard sustainable development as a product of society that is produced through the cooperation of society as a whole to achieve a balance between human development and environmental protection. Given that a society typically acts to maximize benefits while minimizing production inputs through the continuous accumulation of experiences, including both technological innovation and institutional change, we divided the 17 SDGs into three categories: essential needs, expected objectives and governance (Fig.1). We then used this novel ‘matrix’ approach to analyse the complex framework of interactions among the various SDGs, with the overall objective of promoting a coherent policy.

**Figure 1.**
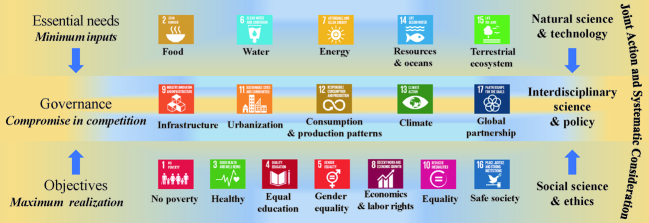
SDG categories: essential needs, governance and objectives. Sustainable development can be seen as a social product. Through appropriate governance, providing maximum outputs with minimal inputs can be realized. For this process to work, minimizing essential needs to improve resource-use efficiency will depend heavily on natural science and technological innovation. Realizing maximum expected goals to better equitably effect the distribution of goods and services will depend more heavily on social science or ethical constraints.

## ELEMENTS OF THE ESSENTIAL-NEEDS CATEGORY ARE CONDITIONAL ON PROGRESS IN SCIENCE AND TECHNOLOGY

The first category—essential needs—represents the basic guarantee of human survival as part of the intrinsic right to realize sustainable development. This category includes food (SDG 2), water (SDG 6) and energy (SDG 7) resources, all of which require ecosystem services provided by land (SDG 15) and the oceans (SDG 14). To achieve sustainable development, these resources must be able to sustain human survival for a long period of time. This requires minimizing their use by reducing resource waste and improving resource-use efficiency—two fundamental requirements for sustainable development in the Anthropocene to both satisfy the needs of the present society and safeguard Earth's life-support system [8].

Scientists and engineers are required to devise innovative solutions to ensure the provision of these essential needs at their lowest optimal rates of consumption and to alleviate the current global-resource crisis by replacing consumption with recycling. Traditional methods of analysis suggest that there is a trade-off between food and energy, especially with regard to water-resource competition [3]. For example, in water-limited regions, industries that require the use of massive amounts of energy not only require water to cool machinery, but also put groundwater and other water resources at risk of contamination, ultimately impacting local food production. Innovation in production technology is the most effective measure by which to overcome such a trade-off between these food and water targets [3]. The development and implementation of these SDGs will rely heavily on scientific contributions at national, regional and global scales; there is also the aspect of the equitable distribution of these essential needs to consider. Such a distribution requires engineering-based improvements in transportation, but also increased assistance from developed to developing countries and regions.

## DEPENDENCE OF EXPECTED-OBJECTIVES ON SOCIAL SCIENCE AND ETHICS

Meeting essential needs guarantees human survival. However, only by meeting the second category of goals, namely expected objectives, can we hope to live prosperous and happy lives. These objectives include living poverty-free (SDG 1) while guaranteeing health (SDG 3), education (SDG 4), gender equality (SDG 5), economic and labour rights (SDG 8) and social equality (SDG 10) and developing a more egalitarian and inclusive society (SDG 16). Unlike the goals in the essential-needs category, which will generally be achieved through an improvement in resource-use efficiency, the key to achieving goals in the expected-objectives category is to reform the manner in which goods and services are distributed. Achieving these goals will rely more on institutional changes, which will necessitate inputs from both the social science and ethics domains [9].

For example, China's 9-year compulsory education system is crucial in achieving SDG 4 (quality education), but further institutional change is required to make higher education more affordable. Similarly, all of the goals in this category must be addressed at the institutional level, which will require great effort from researchers in fields such as sociology.

Diverse geographical, political and economic environments and their relative contexts will present various challenges that restrict the distribution of benefits derived from the SDGs. Therefore, it is necessary to understand the needs and concerns of different groups and individuals, especially in areas subject to ethical constraints. Social disciplines, such as psychology and economics, will undoubtedly play a decisive role in policymaking.

## MEASURES OF GOVERNANCE NECESSITATE EVIDENCE-BASED POLICIES

The third category is governance, which encompasses the effective regulation of competitive relationships and the establishment of equitable rules and systems that will guarantee meeting at least a minimum number of essential needs while maximizing the expected objectives. In this category, building disaster-resistant infrastructure (SDG 9) and sustainable cities and communities (SDG 11) can effectively guarantee the provision of essential needs while maintaining stable economic growth, which is the foundation of social stability. At the same time, establishing responsible production and consumption models (SDG 12) and climate-change control standards (SDG 13) is essential to control emissions from agricultural production and energy consumption. Finally, strengthening global partnerships (SDG 17) can help complement the mutual advantages gained by both developed and developing countries, which will greatly contribute to the implementation of all 17 SDGs.

Given that many environmental and social problems are typically derived from the failure of economic models or management systems [10], appropriate measures are necessary to provide for essential needs and to achieve the desired goals that contribute to human well-being. However, effective governance relies heavily on interdisciplinary guidance, which is to say that inputs from the natural sciences and social sciences are equally important in this regard. For example, in the case of SDG 12 (responsible consumption and production), technological advances could boost the production of clean-energy (green) vehicles. However, the promotion and application of such vehicles will require support from increased public education and preferential policies. Hence, effective governance measures necessitate interdisciplinary collaboration between the various stakeholders, such as scientists, policymakers and entrepreneurs [11].

## DISCUSSION

The relationships between the 17 SDGs are inherently complex. Quantitative-analysis methods, such as the study of correlations and different scenarios, are not always reliable due to the lack of monitoring data for some of the sustainable-development indicators [12]. Addi-tionally, theoretical analyses of the relationships between the various SDGs through the construction of a nexus framework have also been criticized for being subjective, which limits the application of research results [13]. However, viewing the relationships between the SDGs from different perspectives is important in promoting theoretical innovation and adopting SDGs in national policies requires objective evidence [14].

The fundamental difference between our approach and those used in other studies is that we take a holistic view of the relationships among the 17 SDGs, rather than simply analysing their synergies and trade-offs. This approach ensures the comprehensive implementation of the goals and is in contrast to the approach of using a few important indicators, which may not account for the integral application of sustainable development. The purpose of dividing the 17 SDGs into three distinct categories is to simplify the inherently complex relationships between them. This division will also help to improve the management of the various goals, while also promoting interdependent cooperation among different managing bodies and avoiding the narrow perspectives that researchers have used in the past.

Sustainable development is the most important objective of modern times and can only be achieved through a holistic societal approach. Such a broad-based approach is also an effective means to accelerate its realization through a reasonable division of labour and cooperation among managing bodies. Scientific and technological progress can contribute to improving production efficiency to better meet essential needs, providing a greater number of ecosystem services without exceeding the carrying capacity of our planet [15]. Improvements in education, health and social equality (i.e. expected objectives), however, can only be achieved through institutional change, which is strongly influenced by the social sciences. Finally, all of the goals in the governance category are multi-faceted and include meeting the needs of local enterprises and community residents. Moreover, the realization of these goals will help support achieving the objectives in the other two categories.

An in-depth discussion of the synergies and trade-offs among the different SDGs is beyond the scope of this paper, and these issues have been comprehensively discussed by others. Although a change in each goal or indicator will have an impact on other goals or indicators, clustering different goals into groups may facilitate the interactions of various managing bodies and improve the operability of goal management. In addition, this approach could also transform the way we manage SDG relationships from among the 17 individual SDGs to among the three categories, thereby reducing analytical difficulties through simplification. However, in future studies, it is inevitable that the relationships among the 17 goals will have to be analysed separately, which will be a key process in further implementing the SDGs.

## CONCLUDING REMARKS

By grouping the 17 SDGs of the 2030 Agenda for Sustainable Development framework into three categories, we showed that the implementation of the goals within a complex global system is an optimization process governed by a compromise between meeting essential needs and maximizing the realization of expected objectives. Although there are overlaps, trade-offs and synergies among the 17 SDGs, if they are considered holistically, negative externalities among each goal can effectively be excluded. Controlling the consumption of essential needs to their lowest optimal rate is the basic foundation of realizing sustainable development, whereas maximizing expected objectives will satisfy peoples’ material and psychological needs and promote environmental protection. Effective measures of governance are the key to successfully maintaining a balance between meeting essential needs and expected objectives, and each country must find a path of development that suits its own national conditions. Finally, policy coherence, whether horizontal (the interactions between different objectives) or vertical (the interactions between different policy levels), can be ensured by applying this analytical framework to both developed and developing countries.
